# Fitness and fatness in children and adolescents: investigating their role in the association between physical activity and cardiometabolic health

**DOI:** 10.5114/biolsport.2024.129473

**Published:** 2023-06-11

**Authors:** Caroline Brand, Ana Paula Sehn, Camila Felin Fochesatto, Emílio Villa-González, Anelise Reis Gaya, Jane Dagmar Pollo Renner, Alex Ojeda-Aravena, Cézane Priscila Reuter

**Affiliations:** 1 IRyS Group, Physical Education School, Pontificia Universidad Católica de Valparaíso, Valparaíso, Chile; 2 Graduate Program in Health Promotion. University of Santa Cruz do Sul (UNISC). Santa Cruz do Sul, RS, Brazil; 3 Graduate Program in Human Movement Sciences, Federal University of Rio Grande do Sul (UFRGS), Porto Alegre, RS, Brazil; 4 Physical and Sports Education Department. Faculty of Education and Sport Sciences,University of Granada, Spain; 5 School of Physical Education,Physiotherapy and Dance. Graduate Program in Human Movement Sciences, Federal University of Rio Grande do Sul (UFRGS), Porto Alegre, RS, Brazil; 6 Life Sciences Department. Graduate Program in Health Promotion. University of Santa Cruz do Sul (UNISC). Santa Cruz do Sul, RS, Brazil; 7 Physical Education School, Pontificia Universidad Católica de Valparaíso, Valparaíso 2581967, Chile; 8 Health Sciences Department. Graduate Program in Health Promotion. University of Santa Cruz do Sul (UNISC). Santa Cruz do Sul, RS, Brazil

**Keywords:** Cardiorespiratory fitness, Waist circumference, Adiposity, Cardiometabolic risk factors, Youth

## Abstract

To verify the role of the combination of fitness and fatness in the relationship between physical activity (PA) and cardiometabolic risk in children and adolescents. This is a cross-sectional study performed with 2786 children and adolescents (6 to 17 years). Fitness was determined by the cardiorespiratory fitness (CRF) six-minute walking and running test. Waist circumference (WC) was considered a fatness indicator. A selfreported questionnaire was used to determine PA practice, whereas the clustered cardiometabolic risk score (cMetS) was calculated by summing z-scores of triglycerides, total cholesterol/HDL-C ratio, systolic blood pressure, glucose, and WC. Considering the combination of CRF (fitness) and WC (fatness), the following phenotypes were created: Fit/Unfat, Fit/Fat, Unfit/Unfat and Unfit/Fat. Moderation analyses were tested using linear regression models. Significant interactions were found between PA and Unfit/Fat category (β = -0.001; p = 0.001) only for adolescents. The interaction observed in the Unfit/Fat phenotype indicated that adolescents who practise PA for 330 minutes per week presented lower cMetS compared to those who do not practise or practise for 60 minutes respectively. The combination of fitness and fatness moderates the relationship between PA and cardiometabolic risk, suggesting that adolescents, particularly those who are less fit and present high adiposity, should be encouraged to engage in regular PA to improve their metabolic health.

## INTRODUCTION

The onset of cardiometabolic risk factors used to be observed only in adults; however, this scenario has changed, beginning to manifest already during childhood, increasing the risk of developing the cardiometabolic disease later in life [1]. In the context of lifestyle behaviour, the practice of physical activity (PA) brings significant benefits for cardiometabolic health in youth, including decreased low-density lipoprotein cholesterol, insulin resistance, and arterial blood pressure, as well as increased high-density lipoprotein cholesterol (HDL-C) [2, 3]. Similarly, cardiorespiratory fitness (CRF), which is mainly determined by PA habits, is an important health indicator and independent predictor of cardiometabolic risk [4, 5].

In an opposite direction, obesity is a chronic disease associated with the development of many cardiometabolic disorders [6]. The combination of both conditions, fitness and fatness, gives rise to the “fat-but-fit” paradox, which suggests that appropriate levels of CRF could attenuate the deleterious consequences for metabolic health related to an excess of fatness [7]. Therefore, the general belief that being normal weight is equal to being healthy could be wrong since studies have indicated that normal-weight/ unfit subjects could be at a higher risk of developing cardiovascular disease than obese/fit subjects [7, 8]. Some studies reinforce the fat-but-fit paradigm, indicating that CRF could counteract the effect of obesity in cardiometabolic risk factors in children [9], while others shows that both CRF and adiposity are important for having a low cardiometabolic risk [10, 11]. In this context, considering the role of sexual maturation, it is essential due to its influence on physical activity, fitness and cardiometabolic risk factors, in which evidence indicates that early sexual maturation was positively associated with obesity in both girls and boys and also that pubertal stage has an interaction effect on the association of cardiometabolic risk with body mass index [12–14].

Recent studies have suggested that adiposity mediates the association between CRF and cardiometabolic risk [15, 16], which could be related to the correlation between body weight and the computation of CRF. Also, it has been suggested that CRF could play a moderator role in the association between PA and cardiometabolic risk factors [17]. Indeed, these indicators seem to influence the development of cardiometabolic risk in children and adolescents, as well as in the PA levels. However, it is not yet clear how CRF and adiposity interact in this relationship. Based on these assumptions and the strong link among PA, CRF and adiposity, we hypothesized that the combination of fitness and fatness would moderate the association between PA and cardiometabolic risk factors in Brazilian children and adolescents.

Thus, the authors intend to address new evidence in the literature from the perspective of exploring how the combination of fitness and fatness is capable of changing the relationship between PA and cardiometabolic risk factors. Consequently, the present study aimed to verify the role of the combination of fitness and fatness in the relationship between PA and cardiometabolic risk in children and adolescents.

## MATERIALS AND METHODS

### Study design and sample

This cross-sectional study was developed with 2786 children (n = 1170) and adolescents, aged between six and 17 years (mean age 12.17 ± 2.75), from public and private schools from a city located in southern Brazil. Data from the present study belong to a cohort in which the initial sample was recruited in 2004. Students of all regions of the city were considered to calculate the population density of students to be included in the research. Twenty-five schools were randomly selected from fifty schools with 20,380 schoolchildren, considering schools of different regions of the municipality to form a representative sample of this city. All students from the 25 schools were invited to participate in the cohort, which were organized as follows: Phase I (2004–2005), Phase II (2007–2009), Phase III (2011–2012), Phase IV (2014–2015), and Phase V (2016–2017). Data from Phase IV and V were used for the present study. The age range to classify children and adolescents was set according to the Brazilian recommendations [18].

### Procedures

The present study was conducted in accordance with Resolution 466/2012 of the National Council of Health in Brazil, and approved by the research ethics committee at the University of Santa Cruz do Sul (no. 4.278.679). The schoolchildren’s parents or legal guardians signed free and informed consent forms. Data were collected between 2014 and 2017 in the facilities of the University of Santa Cruz do Sul by trained researchers, including physical activity professors, a technical professional in nursing and a pharmacist.

### Evaluations

CRF was assessed by the six-minute walking and running test following the procedures of *Projeto Esporte Brasil* [19]. Schoolchildren should achieve the greatest number of turns, running or walking, in a six-minute period. The test was performed on an outdoor athletic track with demarcations every 10 m to indicate the exact distance (in metres) that was covered. The number of laps completed and the additional distance achieved for the ones unable to complete a full lap at the end of the test were calculated. Thus, CRF was determined by multiplying the number of laps by the metres covered. The sixminute walking and running test is commonly used for the evaluation of Brazilian youth due to ease of application, low cost of material, and the opportunity to evaluate a large number of participants simultaneously [20, 21]. Children and adolescents were classified as Fit and Unfit according to the cut-off points proposed by *Projeto Esporte Brasil* for sex and age [19].

Waist circumference (WC) was evaluated using an inelastic tape with a resolution of 1 mm (Cardiomed), placed at the mid-point between the lower ribs and the iliac crest [23]. WC was classified as Fat or Unfat according to the cut-off points by age and sex proposed by Taylor (2000) [22]. WC was considered the fatness indicator as it is a well-accepted indicator of central adiposity [23]. In addition, it has been suggested that WC presents a stronger association with cardiometabolic risk compared to other anthropometric indicators, such as waist-to-height ratio [24].

Considering the combination of CRF (fitness) and WC (fatness) we created the following categories: Fit/Unfat, Fit/Fat, Unfit/ Unfat and Unfit/Fat.

Weight and height were measured using an anthropometric scale with a coupled stadiometer (Filizola). To determine body mass index (BMI), weight (in kilograms) was divided by height (in square meters).

Self-reported questions were used to determine skin colour and PA practice. The parents of the children aged between 6 and 10 years helped them to fill in the questionnaire. Concerning skin colour, participants were required to indicate one of the following options: white, black, brown/mulatto, indigenous and yellow, as indicated in Brazilian population censuses [25]. PA was evaluated through the following questions: “Do you usually practise any sport/physical activity?” (yes, or no); “How many times a week and hours/minutes per day do you practise this sport/physical activity?” Subsequently, the total minutes per day spent practising sports/physical activities were summed.

Blood samples were collected after 12 hours of fasting to determine triglycerides (TG), total cholesterol (TC), HDL-C, and glucose. Serum samples and commercial kits were used (DiaSys Diagnostic Systems, Germany), performed on Miura 200 automated equipment (I.S.E., Rome, Italy). Systolic blood pressure (SBP) was evaluated by the auscultatory method, using a sphygmomanometer and a stethoscope according to standard procedures [26]. The children/adolescents were supposed to be resting for five minutes prior to the measurement, which was made early in the morning. Each device had three different sized cuffs so that researchers could select the most suitable for each arm circumference. Two measurements on the right arm were made, and the lowest blood pressure was recorded.

The cardiometabolic risk was assessed using a clustered cardiometabolic risk score (cMetS), which was calculated by summing zscores of TG, TC/HDL-C ratio, SBP, and glucose, and dividing the sum by five. Sex- and age-specific standardized z-scores were calculated using international references for each risk factor with the following equation: z-score = ([X –□]/SD), where X is the continuous value observed for the risk factor, □ is the predicted mean calculated for the risk factor using regression equations, and standard deviation (SD) is the standard deviation of the international reference [27]. Before analysis, skewed variables (TC/HDL-C ratio, TG, and WC) were transformed by the natural logarithm.

Sexual maturation was determined according to Tanner’s criteria [28]. The researcher explained the pictures with the different stages of maturation and the schoolchildren were required to indicate the image accordingly to their current stage, considering genital development for boys, breast development for girls and pubic hair for both. Thus, five stages of sexual maturation were considered and subsequently categorized into four: pre-pubertal (stage I), initial development (stage II), continuous maturation (stages III and IV), and matured (stage V). Socioeconomic status was evaluated according to Brazilian Association of Research Companies [29], which considers the head of the household’s educational level and the number of certain items they have (bathrooms, washing machines, car, among others). Each answer was scored and the sum of these scores was used as a measure of the family social class. Subsequently, individuals were classified into three distinct economic classes: low (D-E), medium (C) and high (A-B).

### Statistical Analysis

Descriptive data were presented as mean and standard deviation (continuous variables) and frequencies (categorical variables). All variables were checked to test normality distribution through an exploratory analysis according to box-plot visual inspection and scatterdot graphs to verify the behaviour of variables concerning linearity. Independent two-tailed *t* tests or chi-squared tests were used to examine differences between children and adolescents Moderation analyses were tested through multiple linear regression models using the PROCESS macro for SPSS version 23.0 (IBM Corp). The models were tested as follows: a) Associations between the combination of fitness and fatness categories with cMetS, adjusted for PA; b) Associations between PA and cMetS, adjusted for the combination of fitness and fatness categories; and c) Interactions between the combination of fitness and fatness categories and PA, which consists of the result of the multiplication between the effect of the independent variable (PA) and moderator variable (combination of fitness and fatness categories) in the dependent variable (cMetS).

The PA minutes were categorized into tertiles, provided by the moderation analysis. To test the differences in cardiometabolic risk score according to the combination of fitness and fatness categories in adolescents who practise ≥ 330 minutes of PA a week the ANOVA was used.

All analyses were adjusted for sex, pubertal status, socioeconomic level and skin colour. The probability value p < 0.05 was considered to be significant for all analyses.

Multiple linear regression was used as a statistical test for posthoc sample calculation in the G* Power 3.1 program (Heinrich--Heine-Universität), considering the following parameters: a significance level of α = 0.05, and effect size of 0.02. The number of predictors considered was seven, with a sample size of 2786 children, and test power (1-β) = 0.99.

## RESULTS

A total of 1,170 children (54.0% female) and 1,616 adolescents (59.0% female) participated in the study. Adolescents presented higher mean values of weight, height, BMI, WC, PA, systolic blood pressure, total cholesterol, triglycerides and cMetS compared to children. In addition, 73.6% and 16% of the adolescents were classified in the unhealthy zone for CRF and presented overweight/obesity respectively, while for children 45.6% were classified as unhealthy and 26% were overweight/obese. Concerning the combination of fitness and fatness, 17.8% of the children and 14.1% of the adolescents were classified in the Unfit/Fat category (Table 1).

**TABLE 1:** Characteristics of the sample

	All	Children	Adolescents

	n = 2786	n = 1170	n = 1616

	Mean (SD)			
Age (years)	12.17 (2.75)	9.46 (1.35)	14.13 (1.59)*
Weight (kg)	47.35 (15.11)	36.40 (10.14)	55.28 (13.01)*
Height (meters)	1.51 (0.15)	1.38 (0.10)	1.61 (0.10)*
Body mass index (kg/m^2^)	20.28 (3.98)	18.85 (3.60)	21.32 (3.93)*
PA (minutes/week)	130.14 (188.72)	105.24 (162.51)	148.18 (203.78)*
Systolic blood pressure (mmHg)	105.84 (13.92)	99.39 (12.47)	110.51 (13.03)*
Glucose (md/dL)	88.66 (9.07)	87.22 (9.16)	89.70 (8.86)*
Total cholesterol (md/dL)	161.05 (31.92)	164.61 (31.44)	158.48 (32.02)*
High-density lipoprotein cholesterol (md/dL)	59.20 (11.23)	61.04 (11.40)	57.88 (10.91)*
Total cholesterol/ High-density lipoprotein cholesterol	2.79 (0.66)	2.77 (0.65)	2.81 (0.67)
Triglycerides (md/dL)	74.49 (171.33)	66.98 (30.50)	79.93 (223.33)*
cMetS (z-score)	-0.08 (0.68)	-0.17 (0.70)	-0.02 (-0.67)*

	**n (%)**	**n (%)**	**n (%)**

Sex			
Male	1200 (43.1)	538 (46.0)	662 (41.0)*
Female	1586 (56.9)	632 (54.0)	954 (59.0)
Cardiorespiratory fitness			
Healthy	1064 (38.2)	637 (54.4)	427 (26.4)*
Unhealthy	1722 (61.8)	533 (45.6)	1189 (73.6)
Waist circumference			
Normal weight	2223 (79.8)	866 (74.0)	1357 (84.0)*
Overweight/obese	563 (20.2)	304 (26.0)	259 (16.0)
Combination of fitness and fatness categories			
Fit/Unfat	937 (33.6)	541 (46.20)	396 (24.5)
Fit/Fat	127 (4.6)	96 (8.2)	31 (1.9)
Unfit/Unfat	1286 (46.2)	325 (27.8)	961 (59.5)
Unfit/Fat	436 (15.6)	208 (17.8)	228 (14.1)*
Skin color			
White	2211 (79.4)	970 (82.9)	1241 (76.8)*
Black	203 (7.3)	75 (6.4)	128 (7.9)
Brown/mulatto	335 (12.0)	105 (9.8)	220 (13.6)
Indigenous/yellow	37 (1.3)	10 (0.9)	27 (1.7)
Pubertal status			
Pre-pubertal	583 (20.9)	526 (45.0)	57 (3.5)*
Initial development	639 (22.9)	399 (34.1)	240 (14.9)
Continuous maturation (stage III and IV)	1310 (47.0)	223 (19.1)	1087 (67.3)
Maturated	254 (9.1)	22 (1.9)	232 (14.4)
Socioeconomic status			
Low	1199 (43.1)	488 (41.8)	711 (44)
Medium	1449 (52.0)	624 (53.3)	825 (51.1)
High	138 (5.0)	58 (5.0)	80 (5.0)

SD: Standard deviation; PA: Physical activity; cMetS: Cardiometabolic risk score

* Independent t-test or chi-square for differences between children and adolescents (p < 0.05).

The combination of fitness and fatness in the relationship between PA and cardiometabolic risk in children and adolescents is presented in Table 2. In children, the Fit/Fat and Unfit/Fat categories were positively associated with cMetS; however, no interactions were found. In adolescents, the Fit/Fat, Unfit/Unfat and Unfit/Fat categories were positively associated with cMetS. Also, significant interactions were found between PA and the Unfit/Fat category, indicating that this phenotype is a moderator in the relationship between PA and cMetS.

**FIG. 1:**
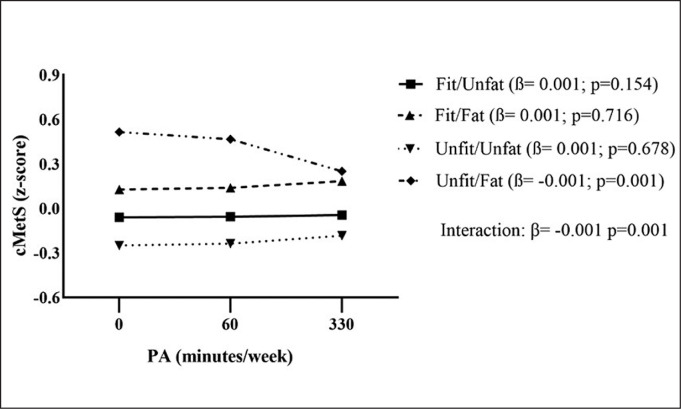
Moderating role of the combination of fitness and fatness in the relationship between physical activity and cardiometabolic rosk in adolescents. Note: PA. Physical activity; cMetS. Cardiometabolic risk score.

For a better comprehension of the interaction observed in adolescents, we present a graphic representation of the moderating role of the combination of fitness and fatness in the relationship between PA and cardiometabolic risk (Figure 1). The interaction observed for Unfit/Fat indicated that for the adolescents who practise PA for 330 minutes per week, the cMetS was lower compared to 0 minutes and 60 minutes of practice. For the other phenotypes, no interactions were found.

**TABLE 2:** Combination of fitness and fatness in the relationship between physical activity and cardiometabolic risk in children and adolescents.

	β	CI (95%)	P

	cMetS		

	Children		
Fit/Unfat	1		
Fit/Fat	0.473	0.292; 0.654	0.001
Unfit/Unfat	0.005	-0.101; 0.110	0.928
Unfit/Fat	0.700	0.577; 0.823	0.001
PA	0.001	-0.001; 0.001	0.630

PA × Fit/Unfat	1		
PA × Fit/Fat	-0.001	-0.001; 0.001	0.813
PA × Unfit/Unfat	-0.001	-0.001; 0.001	0.835
PA × Unfit/Fat	-0.001	-0.001; 0.001	0.113
	
	**Adolescents**		

Fit/Unfat	1
Fit/Fat	0.377	0.080; 0.674	0.013
Unfit/Unfat	0.190	0.091; 0.290	0.001
Unfit/Fat	0.764	0.636; 0.893	0.001

PA	0.001	-0.001; 0.001	0.154
PA × Fit/Unfat	1		
PA × Fit/Fat	0.001	-0.001; 0.001	0.954
PA × Unfit/Unfat	-0.001	-0.001; 0.001	0.370
PA × Unfit/Fat	-0.001	-0.002; -0.001	0.001

PA. Physical activity; cMetS. Cardiometabolic risk score. All analyses were adjusted for sex, pubertal status, socioeconomic level, skin color.

**FIG. 2:**
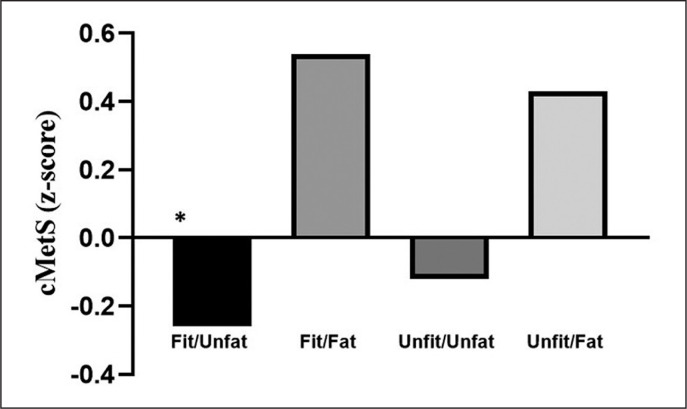
Cardiometabolic risk score according to the combination of fitness and fatness categories in adolescents who practice ≥ 330 minutes of physical activity a week. Note: * Indicates significant compared to Fit/Fat and Unfit/Fat categories.

In addition, we sought to identify the differences in the cardiometabolic risk score according to the combination of fitness and fatness categories in adolescents who practise ≥ 330 minutes of physical activity a week (Figure 2). It was observed that the Fit/Unfat category presented the lowest cMetS compared to Fit/Fat (p = 0.004) and Unfit/Fat (p < 0.001). Also, there was no significant difference between the categories Fit/Unfat and Unfit/Unfat (p = 0.607).

## DISCUSSION

The main findings of the present study indicated that the combination of fitness and fatness is a moderator in the relationship between PA and cardiometabolic risk only in adolescents and not in children. It was observed that for the adolescents with an unhealthy profile, represented by low levels of CRF and high adiposity, the practice of PA for 330 minutes a week may attenuate the cardiometabolic risk score. In addition, for these active adolescents who present a normal weight, being fit does not exert an additional benefit for cardiometabolic health.

There is extensive evidence in the literature concerning the fit-but-fat paradox and its association with cardiometabolic health, indicating controversial results. Some of the studies are in agreement with the paradox [8, 30, 31], while others indicated that CRF is not able to neutralize the negative influence of adiposity on cardiometabolic risk factors [32, 33], showing that CRF is a partial mediator in the association between fatness and cardiometabolic risk factors in European adolescents, although it is not able to completely counteract the negative influence of adiposity on cardiometabolic health. In addition, CRF is considered a prognostic marker of cardiovascular disease, once engaging in regular PA practice is crucial for metabolic health because exercise serves as a powerful stimulator, activating multiple functions at the molecular and cellular levels. This activation can significantly enhance the overall functional capacity of the general population [34]. In obese individuals, the CRF is improved through the regular practice of exercise and demonstrates benefits to metabolic health, independent of weight loss [35]. A recent longitudinal study developed with Brazilian children and adolescents indicated that healthy body composition may exert a stronger protective effect on cardiometabolic health than high levels of CRF [36]. On the other hand, it has been suggested that fitness is a better predictor of cardiovascular disease risk than fatness in adolescents [36, 37].

In this context of modifiable behavioural risk factors, the influence of PA practice must be taken into account. Therefore, the present study sought to go further by determining how PA interacted with the combination of fitness and fatness to exert an influence on a cardiometabolic risk score. To the authors’ knowledge, previous studies have investigated the role of CRF in modifying the association between PA and cardiometabolic risk [17, 38], but these did not investigate the influence of adiposity. The role of adiposity must be taken into account, as healthy body fat seems to be as important as high CRF for metabolic health [36, 37]. In addition, high PA is associated with lower adiposity[39, 40]. Thus, it seems that having low physical fitness, even having a normal weight, may be related to a lower metabolic risk, when the adolescents practised 330 min of PA. Therefore, the ones who present an unhealthy profile but comply with the recommendations of PA practice for Brazilian youth [40] may achieve benefits in cardiometabolic health.

The findings of the present study are partially in agreement with those reported by Brage et al. [38], who found that PA was inversely associated with metabolic risk and interactively associated with physical fitness, indicating that the potential beneficial effect of PA may be more pronounced in children with lower CRF. Similarly, CRF moderated the association between PA with cardiometabolic outcomes; that is, a lower cardiometabolic risk score was observed in children with low CRF, while no associations were observed for children with high levels of CRF [17]. Another study also pointed out that it is necessary to maintain adequate physical fitness and to be physically active for the reduction of abdominal adiposity in overweight/obese children [41].

Regarding obesity and PA, it was observed that PA and adiposity are independently associated with cardiometabolic risk factors, in which individuals with better PA levels and low body fat percentage presented lower cardiometabolic risk. However, when analysing the combination of PA/weight, individuals who are obese and physically active presented higher cardiometabolic risk compared to normal weight and physically inactive individuals, suggesting that adiposity is more important than adequate PA levels for cardiometabolic health [2]. In contrast, another study found that PA practice is essential for health in children and adolescents, especially in overweight/obese individuals, in whom there is a higher risk of developing chronic diseases in adulthood [42]. In general, obese adolescents tend to have lower physical fitness and a lower level of PA compared to non-obese peers [43]. It was also observed that in children and adolescents with low physical conditioning, PA seems to exert a fundamental role in abdominal adiposity [44]. These aspects are associated with many lifestyle behaviours including inadequate sleep, excessive time spent in sedentary behaviour, such as watching television and using electronic devices, unhealthy eating, and less active commuting [45, 46]. It is well known that PA is essential for healthy development in youth, being one important strategy for the prevention and treatment of obesity and its metabolic complications [47]. Moreover, a PA intervention programme is important to improve physical fitness and cardiometabolic risk factors in individuals with overweight and obesity [48]. Indeed, this evidence supports the present findings highlighting the importance of PA, which may attenuate the cardiometabolic risk score in the presence of low levels of CRF and high adiposity. Therefore, the favourable effects on cardiometabolic risk observed in the present study might be explained by the practice of PA, rather than a high CRF per se, as habitual PA is weakly associated with CRF in youth [49].

This study has some important strengths, such as the large sample size composed of Brazilian children and adolescents. It presents new findings regarding the influence of the fat/fit paradox in the association between PA and cardiometabolic risk score, while literature evidence is still controversial about the role of this paradox in cardiometabolic risk. Also, the present study reinforces the importance for unfit/fat adolescents to be physically active, to minimize the risk of developing cardiometabolic disorders in adulthood. However, some limitations should be mentioned. The findings should be cautiously interpreted due to the cross-sectional design which precludes confirmation of cause and effect. The evaluation of PA practice through self-reported questionnaires may lead to underestimating or overestimating data because at the moment it was not possible to evaluate this sample using accelerometry due to the high cost. In addition, we considered only leisure PA, but did not include PA time during physical education classes, and we did not estimate PA intensity. Also, CRF and fatness were assessed using field measurements. Finally, although important potential confounding factors were considered, behavioural factors such as nutritional habits could further confound the observed relationship.

## CONCLUSIONS

In conclusion, this study indicates that the combination of fitness and fatness moderates the relationship between PA and cardiometabolic risk in adolescents. As practical application, the present study suggests that adolescents, particularly those who are less fit and present high adiposity, should be encouraged to engage in regular PA to improve their metabolic health. Thus, it is recommended that future research take into account the difference in ages, due to adolescents being more exposed to the deleterious effects of obesity and low CRF levels on metabolic health.

## References

[1] Halfon N, Verhoef PA, Kuo AA. Childhood Antecedents to Adult Cardiovascular Disease. Pediatr Rev. 2012; 33(2):51–61. 22301031 10.1542/pir.33-2-51

[2] Loprinzi PD, Tudor-Locke C. Weightactivity associations with cardiometabolic risk factors among U.S. youth. Physiol Behav. 2015; 149:165–8. 26056077 10.1016/j.physbeh.2015.06.006

[3] Brand C, Martins CMDL, Lemes VB, Pessoa MLF, Dias AF, Cadore EL, et al. Effects and prevalence of responders after a multicomponent intervention on cardiometabolic risk factors in children and adolescents with overweight/obesity: Action for health study. J Sports Sci. 2020; 38(6):682–91. 32050850 10.1080/02640414.2020.1725384

[4] Andersen LB, Sardinha L, Froberg K, Riddoch CJ, Page AS, Anderssen SA. Fitness, fatness and clustering of cardiovascular risk factors in children from Denmark, Estonia and Portugal: The European Youth Heart Study. Int J Pediatr Obes. 2008; 3(1):58–66. 10.1080/17477160801896366 18278634

[5] Stabelini Neto A, de Campos W, dos Santos GC, Mazzardo Junior O. Metabolic syndrome risk score and time expended in moderate to vigorous physical activity in adolescents. BMC Pediatr. 2014; 14(1):1–6. 24529305 10.1186/1471-2431-14-42 PMC3932015

[6] Stefan N, Schulze MB. Metabolic health and cardiometabolic risk clusters: implications for prediction, prevention, and treatment. Lancet Diabetes Endocrinol. 2023 May. doi: 10.1016/S2213-8587(23)00086-4. 37156256

[7] Ortega FB, Ruiz JR, Labayen I, Lavie CJ, Blair SN. The Fat but Fit paradox: What we know and don’t know about it. Br J Sports Med; 2018; 52(3):151–3. 28583992 10.1136/bjsports-2016-097400

[8] Ortega FB, Lavie CJ, Blair SN. Obesity and cardiovascular disease. Circulation; 2016; 118(11):1752–70. 10.1161/CIRCRESAHA.115.306883 27230640

[9] Pozuelo-Carrascosa DP, Martínez-Vizcaíno V, Torres-Costoso A, Martinez MS, Rodríguez-Gutiérrez E, Garrido-Miguel M. “Fat but Fit” Paradox and Cardiometabolic Risk in Children: The Role of Physical Activity. Child Obes. 2022. doi: 10.1089/chi.2022.0073 35881859

[10] Weisstaub G, Angelica Gonzalez Bravo M, García-Hermoso A, Salazar G, Francisco López-Gil J. Cross-sectional association between physical fitness and cardiometabolic risk in Chilean schoolchildren: the fat but fit paradox. Transl Pediatr. 2022; 11(7):1085–94. 35958004 10.21037/tp-22-25 PMC9360814

[11] Brand C, Gaya ACA, Dias AF, Agostinis--Sobrinho C, Farinha JB, Macedo RCO, et al. The role of adiposity in the relationship between physical fitness with cardiometabolic risk factors, adipocyto-kines and inflammation in children. Sport Sci Health. 2021; 17(1):127–36.

[12] Dai YL, Fu JF, Liang L, Gong CX, Xiong F, Luo FH, et al. Association between obesity and sexual maturation in Chinese children: a multicenter study. Int J Obes. 2014; 38(10):1312–6. 10.1038/ijo.2014.116 25002146

[13] Piola TS, Bacil EDA, Pacífico AB, Camargo EM de, Campos W de. Insufficient physical activity levels and high screen time among adolescents: impact of associated factors. Cien Saude Colet. 2020; 25(7):2803–12. 32667561 10.1590/1413-81232020257.24852018

[14] Chan NP, Choi KC, Nelson EAS, Chan JC, Kong AP. Associations of pubertal stage and body mass index with cardiometabolic risk in Hong Kong Chinese children: A cross-sectional study. BMC Pediatr. 2015; 15(1):136. 26403455 10.1186/s12887-015-0446-0 PMC4582725

[15] Brand C, Reuter CP, Gaya AR, Mota J, Duncan M, Borfe L, et al. Association between cardiorespiratory fitness and cardiometabolic risk factors in Brazilian children and adolescents: the mediating role of obesity parameters. Paediatr Int Child Health. 2021; 41(2):93–102. 33112727 10.1080/20469047.2020.1838758

[16] Pérez-Bey A, Segura-Jiménez V, Fernández-Santos J del R, Esteban-Cornejo I, Gómez-Martínez S, Veiga OL, et al. The influence of cardiorespiratory fitness on clustered cardiovascular disease risk factors and the mediator role of body mass index in youth: The UP&amp; DOWN Study. Pediatr Diabetes. 2018; 20(1):32–40. 30468012 10.1111/pedi.12800

[17] Skrede T, Aadland E, Andersen LB, Stavnsbo M, Anderssen SA, Resaland GK, et al. Does cardiorespiratory fitness moderate the prospective association between physical activity and cardiometabolic risk factors in children? Int J Obes. 2018; 42(5):1029–38. 10.1038/s41366-018-0108-z 29777236

[18] Eisenstein E. Adolescência: definições, conceitose critérios. Adolescência e Saúde. 2005; 2(2):6–7.

[19] Gaya A, Gaya AR. Manual de testes e avaliação: Projeto Esporte Brasil. Porto Alegre: Perfil; 2016; 1–25.

[20] Silveira JF de C, Barbian CD, Burgos LT, Renner JDP, Paiva DN, Reuter CP. Association between the screen time and the cardiorespiratory fitness with the presence of metabolic risk in schoolchildren. Rev Paul de Pediatr. 2020. doi: 10.1590/1984-0462/2020/38/2019134 PMC7274529 32520301

[21] Brand C, Sehn AP, Gaya AR, Mota J, Brazo-Sayavera J, Renner JD, et al. Physical fitness as a moderator in the relationship between adiposity and cardiometabolic risk factors in children and adolescents. J Sports Med Phys Fitness. 2020; 60(12):1567–75. 32614155 10.23736/S0022-4707.20.11130-7

[22] Taylor R, Jones I, Williams S, Goulding A. Evaluation of waist circumference, waist-to-hip ratio, and the conicity index as screening tools for high trunk fat mass, as measured by dual-energy X-ray. Am J Clin Nutr. 2000; 72(2):490–5. 10919946 10.1093/ajcn/72.2.490

[23] Xi B, Zong X, Kelishadi R, Litwin M, Hong YM, Poh BK, et al. International Waist Circumference Percentile Cutoffs for Central Obesity in Children and Adolescents Aged 6 to 18 Years. J Clin Endocrinol Metab. 2020 Apr 1; 105(4):1569–83. 10.1210/clinem/dgz195 PMC7059990 31723976

[24] Agredo-Zúñiga RA, Aguilar-De Plata C, Suárez-Ortegón MF. Waist:height ratio, waist circumference and metabolic syndrome abnormalities in Colombian schooled adolescents: a multivariate analysis considering located adiposity. Br J Nutr. 2015; 114(5):700–5. 26279413 10.1017/S0007114515002275

[25] Instituto Brasileiro de Geografia. Características Étnico-Raciais da População: Classificações e Identidades. Rio de Janeiro; 2013.

[26] Lopes MACQ, Oliveira GMM de, Ribeiro ALP, Pinto FJ, Rey HCV, Zimerman LI, et al. Guideline of the Brazilian society of cardiology on telemedicine in cardiology. Arq Bras Cardiol. 2019; 113:1006–56. 31800728 10.5935/abc.20190205 PMC7020958

[27] Stavnsbo M, Resaland GK, Anderssen SA, Steene-Johannessen J, Domazet SL, Skrede T, et al. Reference values for cardiometabolic risk scores in children and adolescents: Suggesting a common standard. Atherosclerosis. 2018; 278:299–306. 30477756 10.1016/j.atherosclerosis.2018.10.003

[28] Tanner JM. Normal Growth and Techniques of Growth Assessment. 1986. 10.1016/s0300-595x(86)80005-6 3533329

[29] ABEP. ASSOCIAÇÃO BRASILEIRA DE EMPRESAS DE PESQUISAS. Critério Brasil 2015 e Alterações na aplicação do Critério Brasil 2016. Associação Brasileira de Empresas de Pesquisa Critério de classificação econômica Brasil. 2015; 1–6.

[30] Mesa JL, Ruiz JR, Ortega FB, Wärnberg J, González-Lamuño D, Moreno LA, et al. Aerobic physical fitness in relation to blood lipids and fasting glycaemia in adolescents: Influence of weight status. Nutr Metab Cardiovasc Dis. 2006; 16(4):285–93. 16679221 10.1016/j.numecd.2006.02.003

[31] DuBose KD, Eisenmann JC, Donnelly JE. Aerobic fitness attenuates the metabolic syndrome score in normal-weight, at-risk-for-overweight, and overweight children. Pediatrics. 2007; 120(5). 10.1542/peds.2007-0443 17974719

[32] Brand C, Gaya ACA, Dias AF, Agostinis-Sobrinho C, Farinha JB, Macedo RCO, et al. The role of adiposity in the relationship between physical fitness with cardiometabolic risk factors, adipocytokines and inflammation in children. Sport Sci Health. 2021; 17(1):127–36.

[33] Cristi-Montero C, Courel-Ibáñez J, Ortega FB, Castro-Piñero J, Santaliestra-Pasias A, Polito A, et al. Mediation role of cardiorespiratory fitness on the association between fatness and cardiometabolic risk in European adolescents: The HELENA study. J Sport Health Sci. 2021; 10(3):360–7. 33993922 10.1016/j.jshs.2019.08.003 PMC8167318

[34] Wilson MG, Ellison GM, Cable NT. Basic science behind the cardiovascular benefits of exercise. Heart. 2015; 101(10):758–65. 25911667 10.1136/heartjnl-2014-306596

[35] Hamer M, Stamatakis E, Mishra G. Psychological distress, television viewing, and physical activity in children aged 4 to 12 years. Pediatrics. 2009; 123(5):1263–8. 19403489 10.1542/peds.2008-1523

[36] Reuter CP, Brand C, de Castro Silveira JF, de Borba Schneiders L, Renner JDP, Borfe L, et al. Reciprocal longitudinal relationship between fitness, fatness, and metabolic syndrome in brazilian children and adolescents: A 3-year longitudinal study. Pediatr Exerc Sci. 2021; 33(2):74–81. 33857920 10.1123/pes.2020-0197

[37] Buchan DS, Young JD, Boddy LM, Baker JS. Independent associations between cardiorespiratory fitness, waist circumference, BMI, and clustered cardiometabolic risk in adolescents. Am J Hum Biol. 2014; 26(1):29–35. 24136895 10.1002/ajhb.22466

[38] Brage S, Wedderkopp N, Ekelund U, Franks PW, Wareham NJ, Andersen LB, et al. Features of the Metabolic Syndrome Are Associated With Objectively Measured Physical Activity and Fitness in Danish Children: The European Youth Heart Study (EYHS). Diabetes Care. 2004; 27(9):2141–8. 15333475 10.2337/diacare.27.9.2141

[39] Jakicic JM, Davis KK. Obesity and physical activity. Psychiatr Clin N Am. 2011; 34:829–40. 10.1016/j.psc.2011.08.009 22098807

[40] Instituto Brasileiro de Geografia e Estatística. Pesquisa Nacional de Saúde do Escolar. Rio de Janeiro: Instituto Brasileiro de Geografia e Estatística; 2016:1–132.

[41] Medrano M, Arenaza L, Ramírez-Vélez R, Ortega FB, Ruiz JR, Labayen I. Prevalence of responders for hepatic fat, adiposity and liver enzyme levels in response to a lifestyle intervention in children with overweight/obesity: EFIGRO randomized controlled trial. Pediatr Diabetes. 2020; 21(2):215–23. 31778277 10.1111/pedi.12949

[42] Fairclough SJ, Dumuid D, Taylor S, Curry W, McGrane B, Stratton G, et al. Fitness, fatness and the reallocation of time between children’s daily movement behaviours: An analysis of compositional data. Int J Behav Nutr Phys Act. 2017; 14(1):64. 28486972 10.1186/s12966-017-0521-z PMC5424384

[43] Raistenskis J, Sidlauskiene A, Strukcinskiene B, Uğur Baysal S, Buckus R. Physical activity and physical fitness in obese, overweight, and normal-weight children. Turk J Med Sci. 2016; 46(2):443–50. 27511509 10.3906/sag-1411-119

[44] Ortega FB, Ruiz JR, Hurtig-Wennlöf A, Vicente-Rodríguez G, Rizzo NS, Castillo MJ, et al. Cardiovascular fitness modifies the associations between physical activity and abdominal adiposity in children and adolescents: The European youth heart study. Br J Sports Med. 2010; 44(4):256–62. 18463298 10.1136/bjsm.2008.046391

[45] Sehn AP, Gaya AR, Brand C, Dias AF, Kelishadi R, Franke SIR, et al. Combination of sleep duration, TV time and body mass index is associated with cardiometabolic risk moderated by age in youth. J Pediatr Endocrinol Metab. 2021; 34(1):51–8. 33581702 10.1515/jpem-2020-0399

[46] Jia P, Luo M, Li Y, Zheng JS, Xiao Q, Luo J. Fast-food restaurant, unhealthy eating, and childhood obesity: A systematic review and meta-analysis. Obes Rev. 2021; 22(S). 10.1111/obr.12944 PMC7988557 31507064

[47] Hills AP, Andersen LB, Byrne NM. Physical activity and obesity in children. Br J Sports Med. 2011; 45(11):866–70. 21836171 10.1136/bjsports-2011-090199

[48] Vasconcellos F, Seabra A, Katzmarzyk PT, Kraemer-Aguiar LG, Bouskela E, Farinatti P. Physical activity in overweight and obese adolescents: Systematic review of the effects on physical fitness components and cardiovascular risk factors. Vol. 44, Sports Med. 2014; 44(8):1139–52. 24743931 10.1007/s40279-014-0193-7

[49] Ekelund U, Anderssen SA, Froberg K, Sardinha LB, Andersen LB, Brage S. Independent associations of physical activity and cardiorespiratory fitness with metabolic risk factors in children: The European youth heart study. Diabetol. 2007; 50(9):1832–40. 10.1007/s00125-007-0762-5 17641870

